# Climate change-driven range losses among bumblebee species are poised to accelerate

**DOI:** 10.1038/s41598-018-32665-y

**Published:** 2018-10-18

**Authors:** Catherine Sirois-Delisle, Jeremy T. Kerr

**Affiliations:** Canadian Facility for Ecoinformatics Research, Department of Biology, University of Ottawa, 30 Marie-Curie Private, Ottawa, ON K1N 6N5 Canada

## Abstract

Climate change has shaped bee distributions over the past century. Here, we conducted the first species-specific assessment of future climate change impacts on North American bumblebee distributions, using the most recent global change scenarios developed in the Fifth Assessment Report of the Intergovernmental Panel on Climate Change (IPCC). We assessed potential shifts in bumblebee species distributions with models generated using Maxent. We tested different assumptions about bumblebee species’ dispersal capacities, drawing on observed patterns of range shifts to date, dispersal rates observed for bumblebee queens, and, lastly, assuming unlimited dispersal. Models show significant contractions of current ranges even under scenarios in which dispersal rates were high. Results suggest that dispersal rates may not suffice for bumblebees to track climate change as rapidly as required under any IPCC scenario for future climate change. Areas where species losses are projected overlap for many species and climate scenarios, and are concentrated in eastern parts of the continent. Models also show overlap for range expansions across many species, suggesting the presence of “hotspots” where management activities could benefit many species, across all climate scenarios. Broad-scale strategies are likely to be necessary to improve bumblebee conservation prospects under climate change.

## Introduction

Bumblebees (*Bombus* spp.) are important pollinators of many native plant species and agricultural crops, particularly in temperate and high-elevation regions, and are associated with vegetation abundance and diversity^1,2^. Over the past century however, several bumblebee species declined in range and abundance^3,4^. Threats include habitat loss and fragmentation, pesticides, parasites, pathogen spillover, and climate change^5–10^.

Some pollinator species have shifted higher in latitude or elevation in response to periods of rapid climate change^11–13^. However, the majority of bumblebee species have failed to disperse beyond their northern range limits, while suffering losses at their southern range limits^7^. Local extinctions at bumblebees’ southern range limit may be linked to their vulnerability to frequent extreme temperature events under recent climate change^14–16^. Bumblebee decline observed at their historical southern limit, and their failure to track climate change at their northern limit, indicate the potential for increased risks of local extinction under climate change^17,18^.

Research on species’ distributions is critical for informing conservation strategies to mitigate impacts of global climate change on species distributions and range boundaries^19–21^. Species distribution models (SDMs) offer a set of tools to assess species distributions using datasets of georeferenced records, detailed environmental data, and an array of statistical and machine-learning techniques. Maxent^22^ is perhaps the most common approach for SDMs using presence-only data.

The question of whether species’ niche limits, as modeled by SDMs, match their realised range limits is a known challenge in distribution modeling research. In most cases, modeled niche limits encompass observed range limits, beyond which habitat suitability and fitness decline^23,24^. Alternatively, species’ ranges can be distinct from niche limits due to limiting biotic interactions (e.g. antagonist interactions), to physical barriers that limit dispersal, and to low dispersal capacities. As broad-scale environmental changes modify the position of species’ niche limits, an expansion or contraction of species’ range limits over time can occur^23^. When range and niche limits do not coincide, dispersal is key for range-shifts to occur and maintain suitable range where species can persist^23^.

Low rates of species dispersal can amplify mismatches between species’ range limits and niche limits by constraining range expansions at the leading range edge^23,25^. If the trailing edge retracts due to increased mortality rates, while the leading edge shifts slowly or remains stable, ratios of extinction to colonization along range boundaries increase, causing the overall range to shrink^12^. In contrast, net extinction rates decrease if range limits track niche limits where the climate is increasingly suitable^26^. Many species’ survival therefore depends on their capacity to disperse and track suitable conditions under climate change^27–29^.

Investigating bumblebees’ dispersal capacity is important to assess their ability to track suitable environmental conditions and avoid net range losses as climate changes. For bumblebees, dispersal ability (as it translates to changes in species’ geographical ranges) corresponds to the distance that mated queens can travel to establish a new colony^30^. Different bumblebee species have distinct dispersal abilities^31^ but dispersal abilities are unknown for most species and uncertain for most others. Current dispersal estimates range from 3 to 5 km/year^32,33^, increasing to 10 km/year or more for the invasive European species *Bombus terrestris* as it invaded parts of Tasmania^34,35^. Infrequent long distance dispersal events, of unknown frequency and speed, are possible for several bumblebee species^30^. Impacts of dispersal abilities on future bumblebee distribution have never been explicitly addressed at a continental scale for North American bumblebees.

This study asks: how are bumblebee species’ climatically suitable ranges projected to change under different future climate scenarios and with different dispersal abilities? Additionally, are there potential hotspots for the conservation of North American bumblebee species under different climate change scenarios? Potential climate change-related impacts on bumblebee distribution were investigated using a massive dataset of georeferenced observations to generate species distribution models. While relatively few such efforts have yet been made for bees, these models show promise in terms of improving understanding of bumblebee species’ responses to environmental change^14^, and provide insight into species’ performance in new areas based on the projected movement of their climatic niche limits^24^. Study results support previous estimates regarding potentially drastic range losses at the trailing edge of several species as well as their inability to expand under climate change^7^. Our findings highlight the need for prioritizing discussions on assisted colonisation and establishing landscape management of areas where range losses are most likely to halt potentially drastic range contractions.

## Methods

### Maxent modeling

Current and future species-specific distributions based on climatic conditions were projected using Maxent models. These models predict species distributions using existing records and their associate environmental conditions, comparing them to background points, to estimate the edges of species’ tolerances and extrapolate species ranges beyond its known distribution^36^. Maxent was found to capture biologically significant processes as it ranks environmental variables in terms of their importance in delimiting species’ ranges^24,37,38^. This approach has been used to model bumblebee distributions at continental and regional scales^9,14,39–43^. The Maxent algorithm compares conditions at presence and background points – localities where the modeled species has not been sampled – then estimates species distributions based on habitat suitability using the concept of maximum entropy^22,44^. Maxent software was downloaded from http://www.cs.princeton.edu/~schapire/maxent^22^.

### Study area and bumblebee data

**Figure 1:**
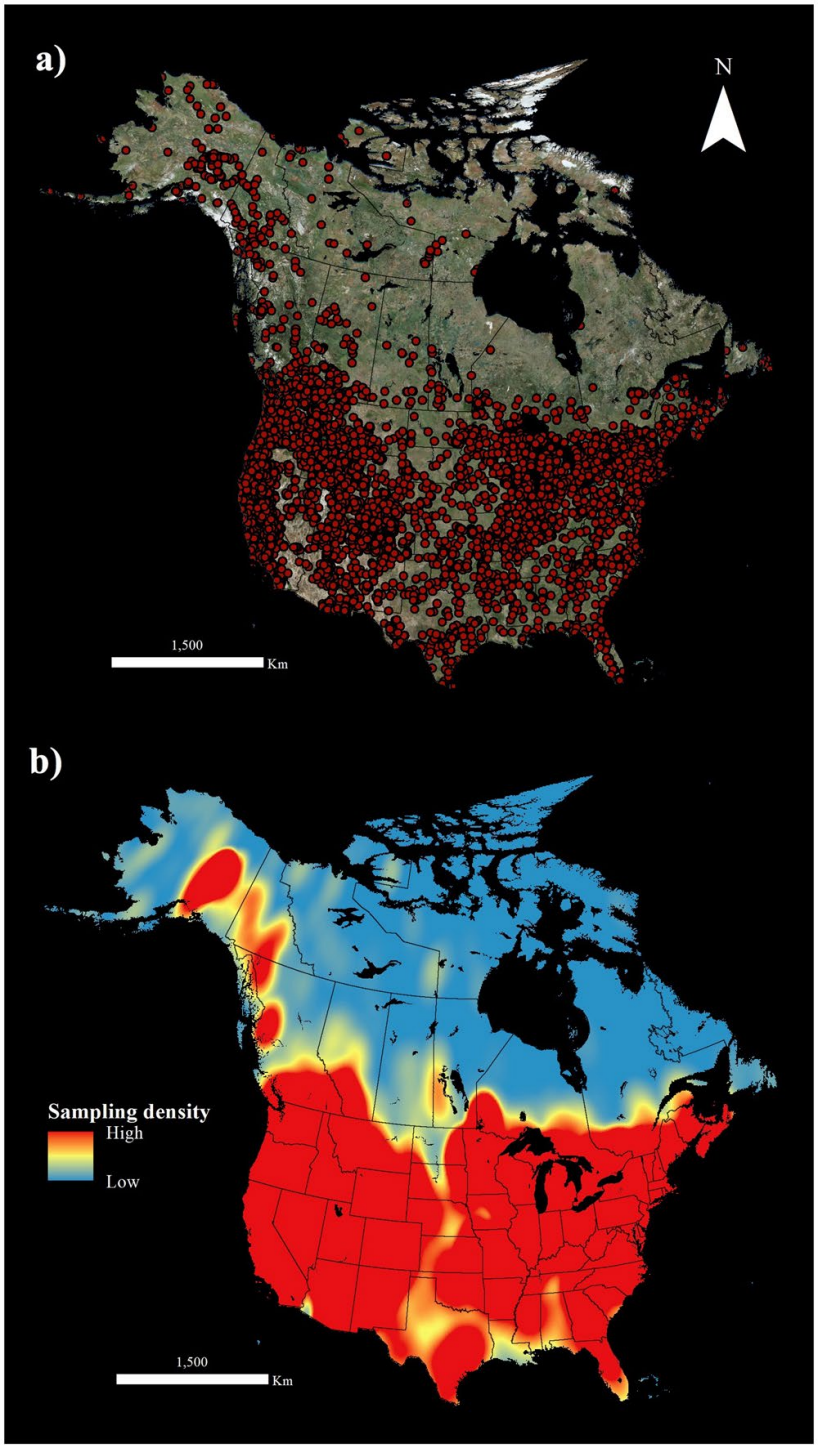
Dataset of georeferenced records for 31 bumblebee species sampled in North America between 1960 and 1990. Data were represented (**a**) by observation points and (**b**) by a heatmap of relative sampling densities.

Primary bumblebee data included 324,502 observations across the North American continent (24 230 000 km2; See Supplementary Information for species list). Georeferenced observations of 31 bumblebee species sampled during years 1960 to 1990 inclusively were extracted, for a total of 19,753 records (Fig. 1). Clusters of observations can cause the model to be overfit, which leads to predicted ranges conforming too narrowly to areas where the species has been observed, an effect that can deceptively increase model performance statistics^45–47^. To address this issue, presence points were spatially rarefied based on climate heterogeneity (i.e. occurrence records were filtered, reducing the dataset to single points within a specified Euclidean distance) to decrease the possibility for sampling bias and autocorrelation^48^. Two heterogeneity classes with minimum distance set to 10 km, and maximum to 15 km were used (classification type set to “Natural breaks”)^40^ to maximize the number of spatially independent points^48^. The rarefied dataset holds 10,628 records for 31 North American bumblebee species, sampled from 1960 to 1990, inclusively.

### Current and future climate data

Current climate data at 5-arcminutes resolution were downloaded from http://www.worldclim.org^49^. From the 19 bioclimatic variables considered for analysis, four were selected to be included in the distribution models of bumblebees for years 1960–1990: annual mean temperature (Bioclim1), temperature seasonality (Bioclim4), annual precipitation (Bioclim12), and precipitation seasonality (Bioclim15). These environmental characteristics are ecologically significant for bumblebees^50^, and measure different aspects of climate to minimize multicollinearity, a recommended approach for variable selection^51^. These variables have been applied to SDMs of bumblebee species in the past^39,40,50^. Further, annual mean temperature and annual precipitation are among the most representative of climate data since they are not derived from other variables^48^.

General circulation models (GCMs) at 5 arc-minutes resolution were obtained from Worldclim and used as future climate data^49^. We used GCMs generated by four major organizations, including the NASA Goddard Institute for Space Sciences, the Meteorological Office Hadley Centre, the University of Tokyo Center for Climate System Research, and the National Center for Atmospheric Research. We investigated future years 2050 (average of 2041 to 2060) and 2070 (average of 2061 to 2080). Each GCM forecasts future climate based on the four Representative Concentration Pathways (RCPs), developed in the IPCC Fifth Assessment Report^19^. RCPs represent different trajectories of atmospheric greenhouse gas (GHG) concentration, where GHG concentration peaks between 2010 and 2020 for RCP2.6, in 2040 for RCP4.5, in 2080 for RCP6.0, or rise continuously to 2100 for RCP8.5.

### Dispersal rates data

Given the importance of dispersal on species persistence under climate change^25^, we evaluated impacts of three different dispersal rates on modeled projections: low, high, and unlimited. Low dispersal was assumed to be negligible, where only retractions from currently suitable ranges are possible, as observed for the majority of surveyed species at their northern range limit^7^. High dispersal was set to 10 km/year, which corresponds to the highest recorded dispersal rate of invasive populations of *B*. *terrestris*^30,35^. Unlimited dispersal was meaningful to reveal changes in the position of species’ climatic niche, regardless of dispersal ability^50^.

### Maxent modeling settings

Model settings accounted for uncertainty in future climate change and data limitations. Species-specific bias files were created on ArcGIS with minimum convex polygons around occurrence points, to decrease the probability of including suitable but uncolonised localities in the model as background points, which can lead to commission errors^52,53^. Models were replicated using 10-fold cross-validation to assess model fit and uncertainties in spatial predictions^51^. Occurrence data was randomly and equally split into 10 folds, creating 10 models, where each run uses a different single fold for model validation. While generally recommended^51^, we are aware of K-fold cross validation limitations, such as the potential for spatial correlation between folds, which would overestimate model performance and underestimate the standard error of predictions^53^. The default regularization multiplier was used, which has been used to model multiple bumblebee species’ distributions successfully^39,41,43^. Clamping was used, setting values outside the training data within projections to the maximum training range. This is useful for projecting into the future since bumblebee species’ survival in future novel climatic conditions is unknown^44^. The logistic output format was chosen to obtain a relative probability of occurrence and compare models for several species^44^.

### Evaluating bumblebee range changes

Species-specific logistic probabilities of occurrence were averaged across GCMs, for each RCP scenario and time period, using ArcGIS 10.2. To obtain binary presence-absence maps, we used the probability of occurrence threshold that maximized the True Skill Statistic (TSS) for each species, as recommended^51^. Dispersal scenarios were then applied to binary maps by clipping modeled projections to 0 km/year, 10 km/year, and unlimited dispersal rates from current distributions. Maps were summed across all species for the four RCPs, three dispersal scenarios, and two time periods to generate a total of 24 species richness maps. Changes in species’ suitable ranges across North America were plotted using R statistical software. Differences between current suitable range and future scenarios revealed areas projected to gain or lose species, which were then overlapped across all species and RCPs to identify regions where they are concentrated. These overlaps were examined using land-use data. Land use data for 2016 were downloaded from the History Database of the Global Environment (HYDE) at http://themasites.pbl.nl/tridion/en/themasites/hyde^54^. The HYDE dataset combines satellite data and statistics of world population, cropland and pasture in 5 arc-minutes resolution maps. Richness changes were analysed for agricultural areas, but we caution that comparative trends in richness change within agricultural areas could be overstated, since our dataset focuses on species whose ranges overlap those areas.

### Predictive accuracy

We used two measures of model evaluation. Common methods include the Area Under the Receiver Operator Characteristic Curve (AUC)^55^, and the True Skill Statistic (TSS)^56^. The threshold independent AUC is calculated by default in Maxent models. If AUC < 0.75, model predictions are not significant and cannot be interpreted^57^. When AUC ≥ 0.75, models were considered to successfully explain present distribution^58^. TSS is threshold dependent, as it is based on binary presence-absence maps. The threshold that yields the highest TSS was selected to produce binary maps^56^. The formula to calculate TSS is:

$$({\rm{TSS}}={\rm{sensitivity}}+{\rm{specificity}}-1),$$

where sensitivity corresponds to the true positive rate and specificity is the true negative function^59^, considering that Maxent models the absences. If TSS ≥ 0.4, models perform better than random predictions^60^. TSS was shown to be a reliable measure of predictive ability^56^ since it does not depend on the size of the validation dataset like other statistics^61^.

## Results

### Predictive accuracy

Predictive accuracy of Maxent models was generally high, indicating useful models^57^. Models produced acceptable AUC values (AUC ≥ 0.75) and TSS values (TSS ≥ 0.4) for all species except *Bombus centralis*, which was consequently removed from the analysis. Mean AUC for all species was 0.85, and mean TSS was 0.62 (See Supplementary Information for species-specific model accuracy statistics).

### Evaluating bumblebee range changes

**Figure 2:**
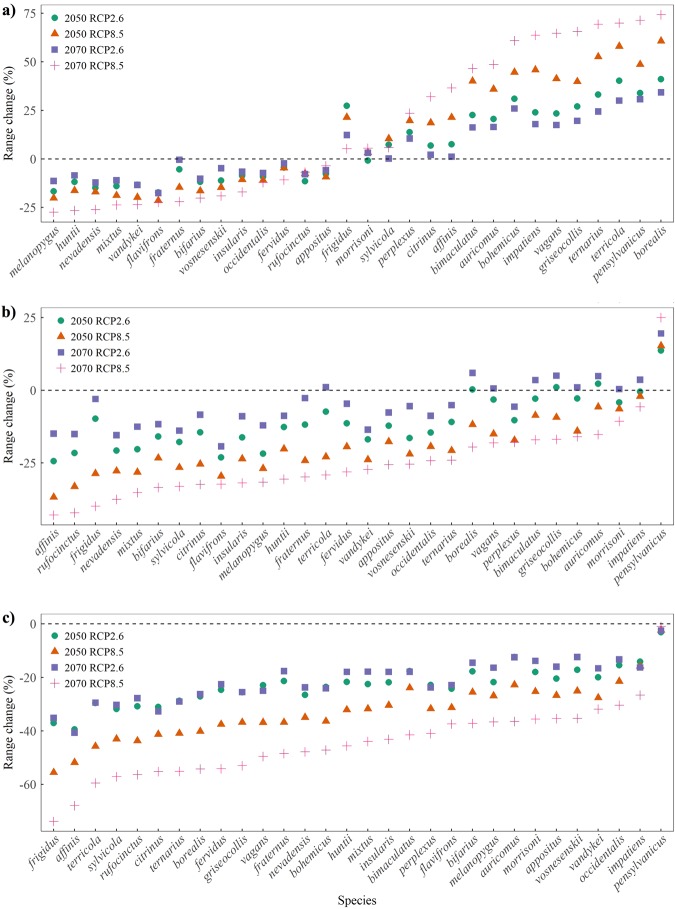
Range changes based on Maxent models for 30 North American bumblebee species between baseline (1960–1990) and future climate projections (RCP2.6 and RCP8.5) in 2050 and 2070, assuming (**a**) unlimited dispersal, (**b**) high dispersal (10 km/year), or (**c**) negligible dispersal ability (0 km/year). Results were ordered by range change (%) under the RCP8.5 scenario in year 2070.

Models showed range expansions for about half of bumblebee species under unlimited dispersal scenarios in both 2050 and 2070 (Fig. 2a). Between 0–13% of species expanded in range, varying with RCP scenarios, when dispersal input was closer to realistic abilities (10 km/year) by 2050. This proportion increased to 0–30% by 2070, but most expansions were only found in a single climate scenario (RCP2.6) (Fig. 2b). Only *Bombus pensylvanicus* was projected to expand in all RCPs, in both 2050 and 2070 when dispersal was constrained to 10 km/year. Expansions were not possible restricting species from dispersal in future years (Fig. 2c).

Even assuming unlimited dispersal, nearly half of bumblebee species were projected to face significant range losses by 2050 (Fig. 2a). Constraining dispersal to 10 km/year aggravated range losses of 87–97% of modeled species’ current range by 2050, increasing to 70–97% by 2070, depending on the RCP outcome (Fig. 2b). Under no dispersal, models showed losses of over half of current suitable range by 2050 for some species, rising to nearly three quarters by 2070. Only *Bombus pensylvanicus* maintained relatively stable distributions in all RCP scenarios, under this dispersal scenario, for both 2050 and 2070 (Fig. 2c). The discrepancy of range changes between different RCPs was far more significant in 2070; mean standard deviation in suitable range changes doubled from 2050 to 2070 under all three dispersal scenarios.

### Species distribution: hotspots and species loss overlays

**Figure 3:**
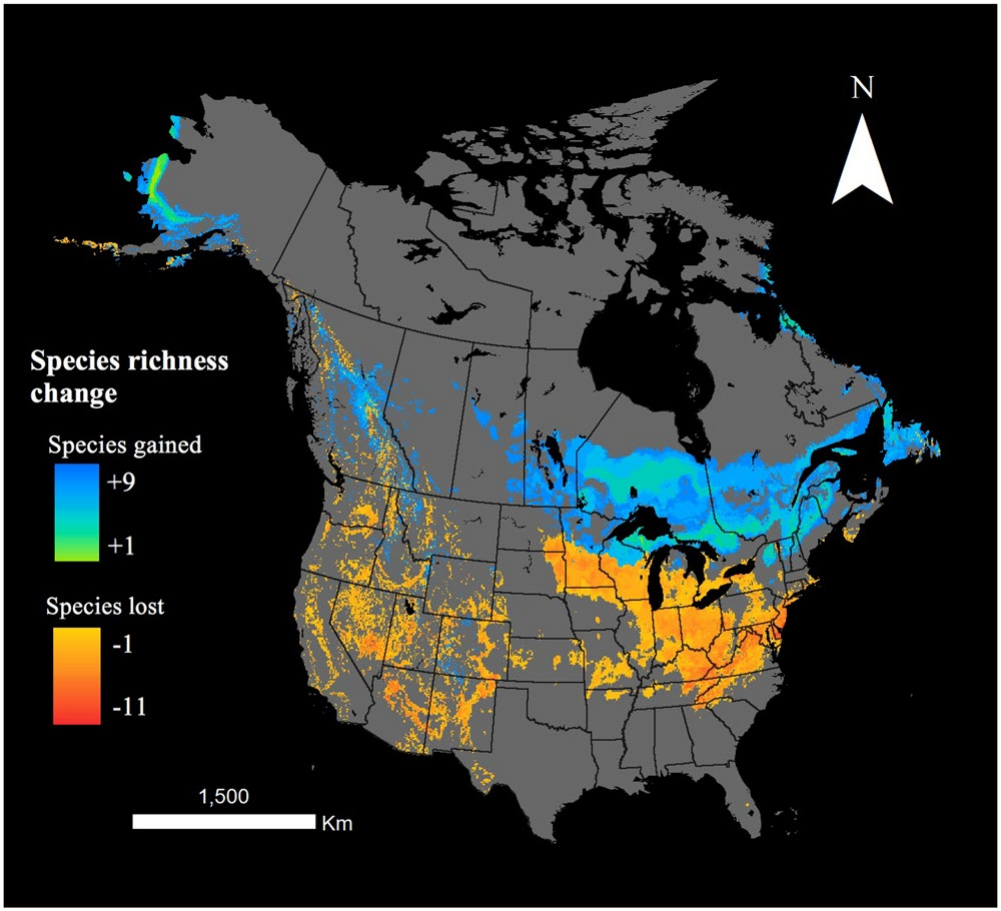
Overlays across all species future projections and RCP scenarios likely to gain suitable range for multiple species, and lose multiple species by 2070.

Regions where multiple bumblebee species’ ranges were projected to expand in 2070 were concentrated, and generally not located beyond the northern limit of densely sampled observations. These hotspots were mapped and overlapped across all RCP scenarios to identify areas suitable for new species in the future regardless of climate trajectory (Fig. 3). By 2070, these areas were large, ranging from 11 419 km^2^ (9 species) to 2 447 283 km^2^ (3 species). They are mostly found in Ontario, Quebec and northern parts of Michigan, while generally less prevalent in western parts of the continent. Ecoregion types associated with hotspots are mainly mixed wood plains, mixed wood shield and softwood shield (WWF 2016). Less than 25% of these areas are currently developed or disturbed for agricultural purposes, according to the HYDE 2016 land use dataset.

Areas where range losses overlapped among species were relatively concentrated (Fig. 3). By 2070, these areas ranged from 75 km^2^ (11 species) to 2 173 231 km^2^ (3 species). Losses primarily occurred in Tennessee, Kentucky, Virginia, Maryland and Delaware, and are essentially dominated by Ozark, Ouachita-Appalachian Forests, south-eastern USA plains, central USA plains, and mixed wood plains ecoregions (WWF 2016). These ecoregions are generally dominated by agricultural lands, developed land, or by mosaics of forest, pasture, cropland, developed land, and/or wetlands^62^, and are characterized by high intensity land uses in the form of crop and grazing lands.

### Species richness losses in agricultural areas

**Figure 4:**
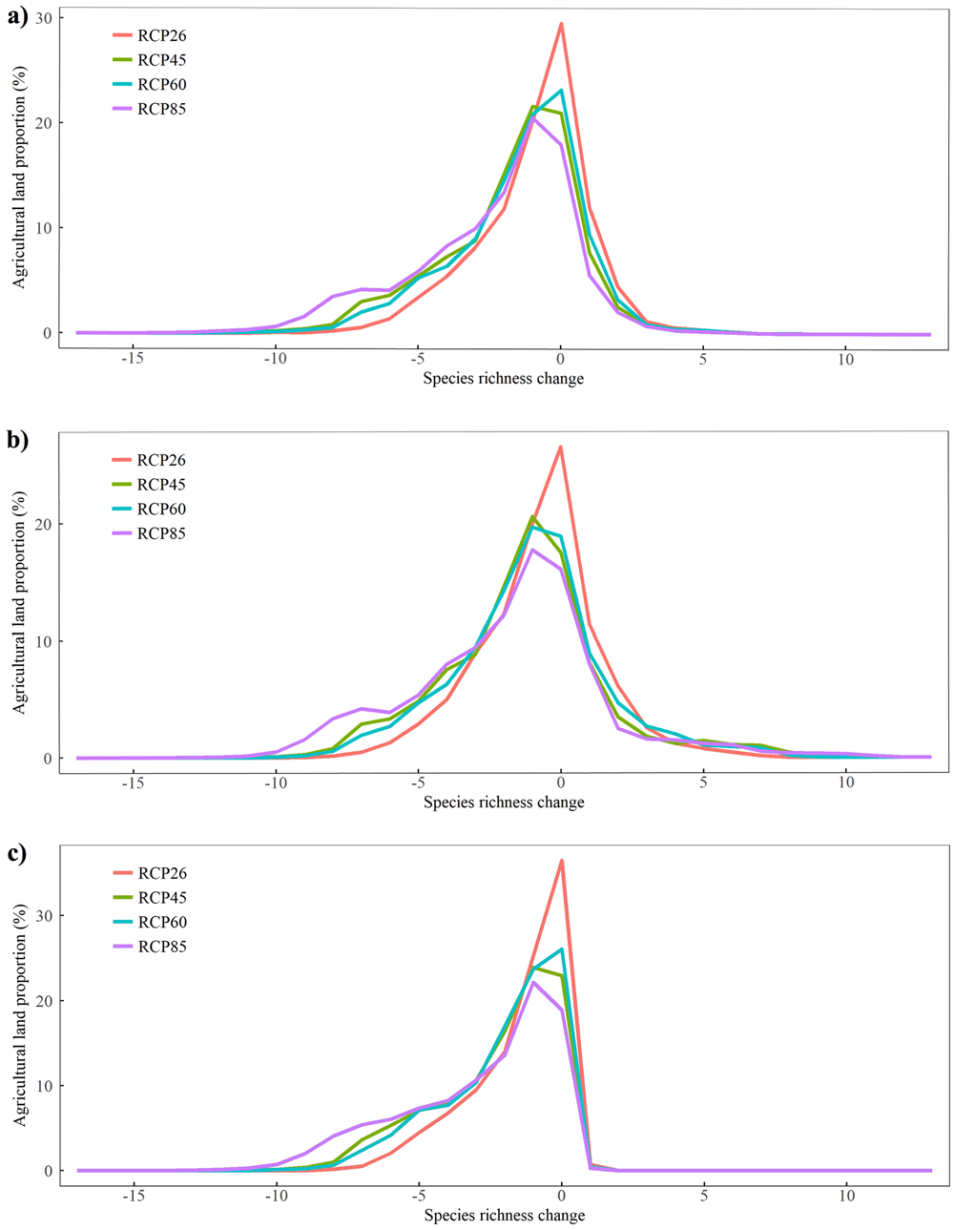
Histograms of species richness change in agricultural areas of North America based on three dispersal assumptions; (**a**) unlimited dispersal, (**b**) high dispersal and (**c**) no dispersal for all 30 modeled bumblebee species. The Y axis represents the percentage of all North American agricultural areas based on the HYDE (2016) land use dataset. The X axis corresponds to species richness changes (unit: number of species).

Species richness decline is disproportionately severe in agricultural areas. Within agricultural areas, most areas lost species and few gained species (Fig. 4). The least severe climate change scenario (RCP2.6) is distinct from the other three scenarios (RCP4.5, RCP6.0, RCP8.5) in which species richness loss was consistently superior. Dispersal scenarios did not affect this outcome, although slower dispersal led to aggravated species richness decline (Fig. 4).

## Discussion

All model results, regardless of climate change scenarios or assumptions about dispersal capacity, suggest that significant declines of bumblebee species across much of North America are likely. Models reveal large range losses even in scenarios where dispersal abilities are estimated using the highest recorded dispersal rates for an invasive bumblebee species (10 km/year)^30^. Across a range of realistic dispersal rates, few bumblebee species are likely to maintain stable geographical range sizes, let alone track warming rapidly into historically-unoccupied areas beyond species’ current ranges. Even pollinators known to disperse at especially high rates under climate change, such as some butterfly species, have accumulated substantial climate debt^29,63^ – the lag between species actual colonization of new areas and the rate required given the pace of climate change –, a prospect our results suggest is likely to be especially severe among bumblebee species.

Even assuming unlimited dispersal, about half of modeled bumblebee species’ ranges are projected to decline in all future climate scenarios. Large interspecies variation arose because of differences in spatial shifts of species’ climatically suitable range into new areas and the extent to which areas in their current ranges were forecasted to become unsuitable. Range losses were focused in regions that are also strongly disturbed by various anthropogenic activities (e.g. agricultural and developed land). Areas where multiple species expanded (i.e. hotspots) were concentrated in regions poleward of current distributions, where developed or agricultural lands are limited^62^. The extent of hotspots was largest in areas east of the Rockies where the geographical trajectories of anticipated climate changes are mostly northward^64^. The relatively uniform topography of eastern regions generates weak climate gradients, so large latitudinal shifts are necessary for species to track changing climatic conditions^17,65^. Comparatively smaller distances must be traveled to track similar climatic changes within mountainous areas^13^. Under high or unlimited dispersal, range changes are more accentuated in 2050 than 2070 for some species, but only under strong climate mitigation (RCP2.6). The relatively low losses at most species’ trailing edge of this moderate RCP scenario were compensated more easily at the leading edge in 2070 while losses were not significantly worsened. Species-specific traits, however, ultimately determine species’ capacities to reach newly suitable areas in the future, regardless of environmental or anthropogenic barriers^13,17^, and may explain variation associated with bumblebees’ realised responses to climate change^66^. Bumblebees’ ability to reach and persist in new areas, necessary to maintain their range over time, is particularly important within agricultural areas where ecosystem services hold important economic and ecological value for human populations^4^.

Widespread species richness declines were predicted for agricultural areas of North America, where substantial changes in pollination networks are already expected due to known threats like land-use change, various chemicals used by the agro-industry, parasites and pathogens^67^. Bumblebee richness decline is expected to impair pollination services^4^. Subsequent negative impacts on food yields^68^ and on human welfare are also likely^69^. Changes in landscape connectivity and host plant abundance could drastically alter expected impacts^4^. Our results show that several species could be vulnerable to climate change in agricultural areas across the North American continent. Several species seem likely to require management intervention with a broad-scale perspective, to prevent potentially synergistic effects of agricultural practices and climate change on bumblebee decline.

Our approach is conservative in its assessment of potential range losses among bumblebees by excluding measures of potential land-use impacts from models. Including land-use data at appropriate spatial scale in Maxent modeling processes would probably assess species niches more broadly than purely on climatic grounds^70^. However, historical land-use data for North America is coarse thematically, temporally, and spatially. Detailed land-use models that address these limitations of resolution for the future do not exist but, regardless, are necessarily highly uncertain relative to climate models^71^.

Interactions between climate change and land-use change are expected to exacerbate species range losses in the future^72^, suggesting that future range declines may be even steeper than those reported here. Land-use change may hinder species dispersal under climate change while local climate changes may similarly interact with, and impair, species’ responses to land-use changes^73^. Land-use changes are likely to contribute especially to widespread biodiversity losses under strong climate mitigation scenarios (RCP2.6) due to rapid expansions of infrastructure like biofuel plantations^72^. Conversely, climate changes are likely to exert more severe negative impacts than land use changes under RCP8.5^72^, which most closely reflects the trajectory of current emissions^64,74^. Consequently, range declines we project in this study likely provide an optimistic view of species realised future ranges, emphasizing the urgent need for effective bumblebee conservation strategies.

Less than 1% of areas we identify as hotspots for bumblebee range expansion are currently protected, according to the World Database on Protected Areas (WDPA). Recent efforts to rapidly expand protected area networks in Canada to improve prospects for conservation are likely to benefit many taxa, including bumblebees. Accounting for ecosystem services and species’ range dynamics consequent to climate changes are central to these efforts^75^.

Findings support the need for effective mitigation strategies that could benefit multiple species simultaneously, and aim to increase the likelihood of successful colonizations in areas beyond those occupied historically. Our data revealed relatively large and concentrated hotspots overlapping across climate scenarios, supporting broad-scale management plans for multiple bumblebee species. These hotspots include candidate sites where assisted colonisation efforts could be concentrated and would benefit several species^76^. Such efforts might help species maintain broader geographical ranges than would otherwise be possible, reducing the prospects of species extinctions and erosion of pollination services associated with rapid climate change^26,28,77,78^. Assisted colonisation precludes introductions of species across biogeographical boundaries^28,79^, avoiding the creation of non-analog ecological communities and should account for risks of introducing novel genotypes of pathogens to new areas. Bumblebees’ high risk of decline under climate change, the relative practicality of translocation, and manageable costs of relocating small numbers of fertilized queens in the spring, together, suggest that assisted colonisation should be evaluated alongside conventional conservation strategies focusing on habitat characteristics^28^. Assisted colonisation remains controversial on ethical grounds and due to potential risks of relocated species becoming locally invasive^26,80–82^.

Mitigation strategies can be directed toward areas where climate changes poses the greatest risks to bumblebee populations^8,83^. Landscape management to protect habitat across species’ dispersal pathways or corridors could facilitate range shifts^84,85^. Species rely on habitat availability beyond their historical range to disperse and track shifting climate conditions^86^. Further, habitat management in areas where climate-driven species losses are concentrated could slow range losses. Among species included in this study, such areas are subject to intensive land uses^62^. Habitat management can decrease pressures on bumblebees^87^, reducing the impacts of other threats and providing micro-refugia enabling bees to escape thermal extremes^50^. Microclimatic heterogeneity improves probabilities of survival at the edge of species’ distributions^88^ where they are most vulnerable to other threats^89^.

Even though species distribution models mirror mechanisms that modulate species distribution and hold ecological significance to predict past and future distribution^37^, they make a number of simplifying assumptions^90,91^. Models assume that the selected environmental variables are the main contributors to the position of species’ ranges, but other biotic and abiotic interactions have an effect on their distributions^92^. SDMs can also underestimate species’ niches because they assume that all suitable habitat is colonised, even though biotic or anthropogenic barriers can prevent species’ ranges from reaching equilibrium with environmental conditions^90^. Impacts of these assumptions are alleviated at the continental scale because climate is a main contributing factor to species distributions at this scale^21^. Further, factors like pesticides and land use change have not been observed to interfere with bumblebee range shifts in Europe and North America to date at those broad spatial extents^7^. SDMs can produce useful insights into how environmental factors shape species’ geographical ranges independently of complex species interactions or contingent factors^50^, but testing models over time to test their validity after periods of observed climate change is important^93–95^.

Bumblebee species’ dispersal capacities and generalized losses of climatically suitable areas make it likely that most bumblebee species included here will see substantial range losses in the coming decades. Widespread range losses for North American bumblebees seem likely even when assuming improbably rapid dispersal that has only been observed in the most extreme instances of *Bombus terrestris* invasion of new environments. Bumblebee species are particularly effective pollinators^4^ and their projected declines are especially pronounced in agricultural areas, so global change-induced erosion of those services could have both ecological and pronounced economic significance^96,97^. Discussions around whether, where, and for which species assisted colonisation should be considered are warranted, as are expanded efforts to manage habitats to retain species in areas where climatic conditions are likely to become less suitable in the near future.

## Electronic supplementary material

Supplementary Information

## Data Availability

The datasets generated during the current study are available from the corresponding author on request or from http://www.macroecology.ca/.
