# Supplementary material for: Climate change-driven range losses among bumblebee species are poised to accelerate
Source: Sci Rep. 2018 Oct 18;8:14464. doi: 10.1038/s41598-018-32665-y (PMC6194031; doi:10.1038/s41598-018-32665-y)
Supplement: Supplementary file 1 — Supplementary Information [file 41598_2018_32665_MOESM1_ESM.pdf]

Supplementary Information for

Climate change-driven range losses among bumblebee species are poised to accelerate

CATHERINE SIROIS-DELISLE and JEREMY KERR

University of Ottawa, Department of Biology, 30 Marie-Curie Private, Ottawa ON K1N 6N5, Canada

This PDF file includes:

Supplementary Design and Methodology
Supplementary Results: Figures S1 to S4

Supplementary Design and Methodology

Bumblebee data

To obtain our final dataset (Dataset 1) from the primary bumblebee data, preparation first involved the removal of inadequate records, and the extraction of the records of interest. Records holding incomplete information for species identification, locality or year, or inaccurate georeferenced points, and duplicate records, were removed. Points less than 2500m from shoreline were moved to the closest point on the coast. Georeferencing data from GeoNames (http://geonames.org; Creative Commons Attribution 3.0 License) were used for GBIF points lacking geographical coordinates. A total of 19,753 records for 31 bumblebee species were extracted. The following list enumerates bumblebee species sampled across North America between 1901 and 2010, of sufficient reliability and sampling effort for data analysis.

- *Bombus affinis*
- *Bombus appositus*
- *Bombus auricomus*
- *Bombus bifarius*
- *Bombus bimaculatus*
- *Bombus bohemicus*
- *Bombus borealis*
- *Bombus centralis*
- *Bombus citrinus*
- *Bombus fervidus*
- *Bombus flavifrons*
- *Bombus fraternus*
- *Bombus frigidus*
- *Bombus griseocollis*
- *Bombus huntii*
- *Bombus impatiens*
- *Bombus insularis*
- *Bombus melanopygus*
- *Bombus mixtus*
- *Bombus morrisoni*
- *Bombus nevadensis*
- *Bombus occidentalis*
- *Bombus pensylvanicus*
- *Bombus perplexus*
- *Bombus rufocinctus*
- *Bombus sylvicola*
- *Bombus ternarius*
- *Bombus terricola*
- *Bombus vagans*
- *Bombus vandykei*
- *Bombus vosnesenskii*

Climate data

Climate data were obtained from worldclim.org. Climate data were generated using ANUSPLIN modeling software except for temperature and precipitation seasonality, which correspond to the standard deviation of monthly values, expressed as a percentage of the mean for those estimates[1].

We investigated four scenarios (RCP2.6, RCP4.5, RCP6.0 and RCP8.5). According to the most recent IPCC report (2014), temperature rise for most optimistic scenario (RCP2.6) is *likely* to be between 0.3 – 1.7°C by 2100, compared to pre-industrial temperatures (1850 – 1900). This scenario includes the adoption of stringent climate policy and mitigation measures. RCP4.5 projects a rise of 1.1 – 2.6°C (*likely* below 2.0°C), also achieved through the adoption of new climate policies. A rise of 1.4 – 3.1°C for RCP6.0 or of 2.6 – 4.8°C for the most pessimistic scenario (RCP8.5) are projected if no climate mitigation measures are adopted[2]. Across all scenarios, it is *very likely* that hot temperature extremes will be longer lasting and more frequent, while cold extremes will be less prevalent throughout most terrestrial habitats. Precipitation patterns are projected to change drastically across all scenarios. For RCP8.5, they are likely to increase under high latitudes, the equatorial pacific, and mid-latitude wet regions, and decrease under mid-latitude and subtropical dry regions[2].

We used the four RCPs as described above into future years 2050 (average of 2041 to 2060) as well as 2070 (average of 2061 to 2080), as modeled under the following general circulation models (GCMs):

- GISS-E2-R model from the NASA Goddard Institute for Space Sciences;
- HadGEM2-AO model from the Meteorological Office Hadley Centre;
- MIROC5 model from the University of Tokyo Center for Climate System Research;
- CCSM4 model from the National Center for Atmospheric Research.

Supplementary results: Figures S1 to S4

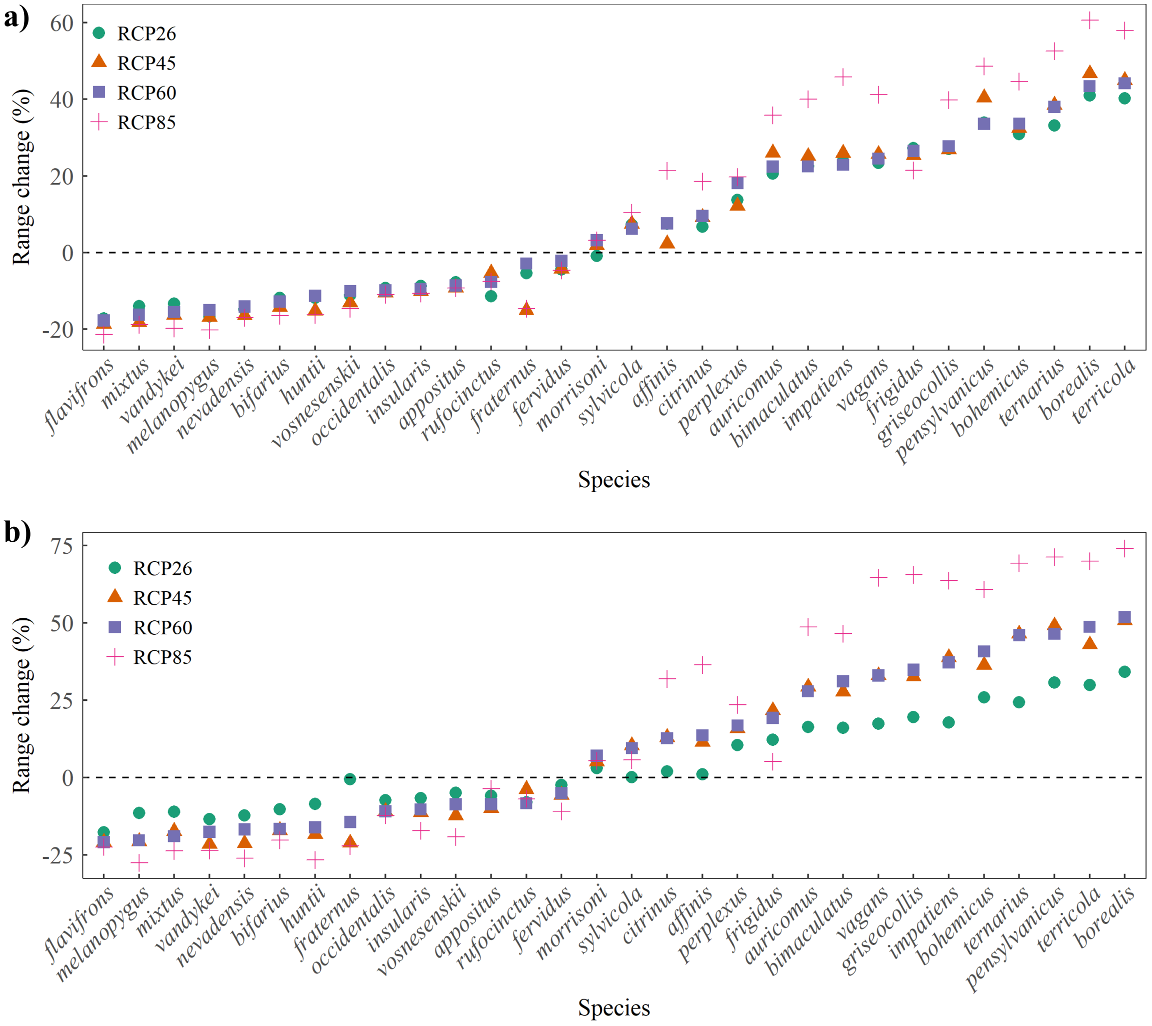

Figure S1: Range changes based on maxent models for 30 North American bumblebee species between baseline (1960-1990) and future projections of years (a) 2050 and (b) 2070, assuming unlimited dispersal ability. Results were ordered by range change (%) under the RCP6.0 scenario. The four RCPs represent a range of possible radiative forcing increases in W/m^2 (+2.6, +4.5, +6.0, +8.5) between pre-industrial values and the year 2100.

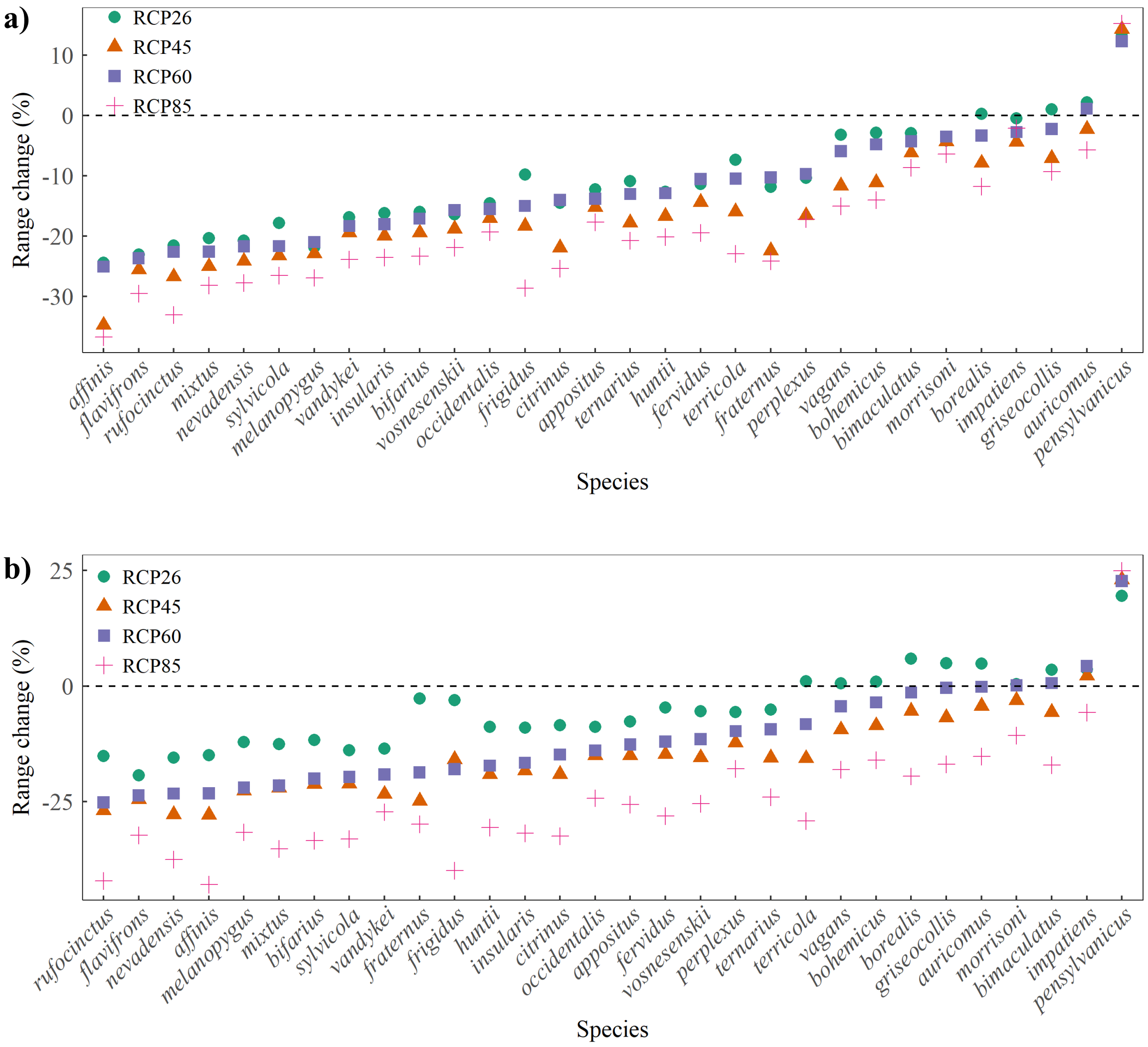

Figure S2: Range changes based on maxent models for 30 North American bumblebee species between baseline (1960-1990) and future projections of years (a) 2050 and (b) 2070, assuming a high dispersal rate (10 km/year). Results were ordered by range change (%) under the RCP6.0 scenario. The four RCPs represent a range of possible radiative forcing increases in W/m^2 (+2.6, +4.5, +6.0, +8.5) between pre-industrial values and the year 2100.

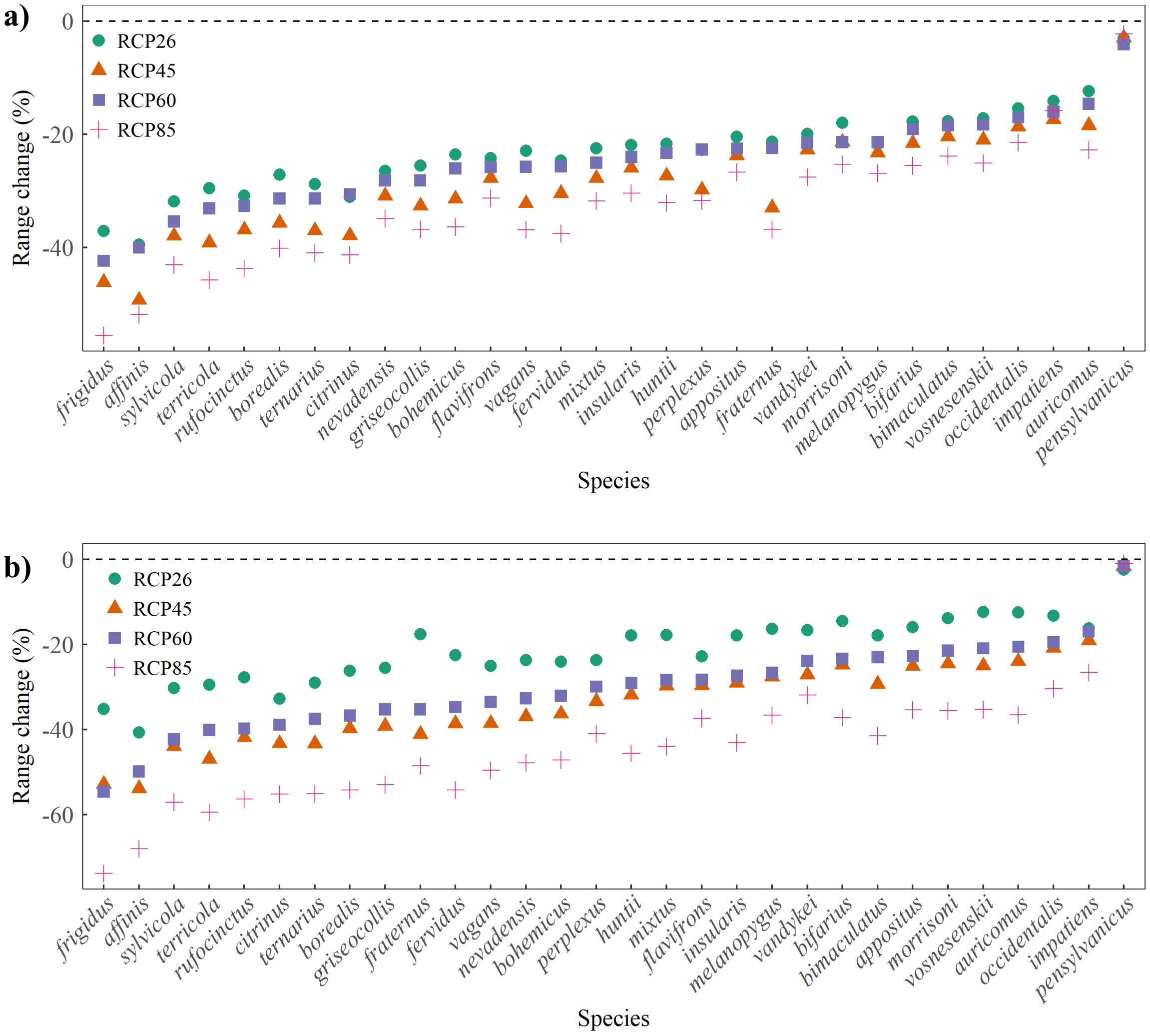

Figure S3: Range changes based on maxent models for 30 North American bumblebee species between baseline (1960-1990) and future projections of years (a) 2050 and (b) 2070, assuming a dispersal rate of 0 km/year. Results were ordered by range change (%) under the RCP6.0 scenario. The four RCPs represent a range of possible radiative forcing increases in W/m^2 (+2.6, +4.5, +6.0, +8.5) between pre-industrial values and the year 2100.

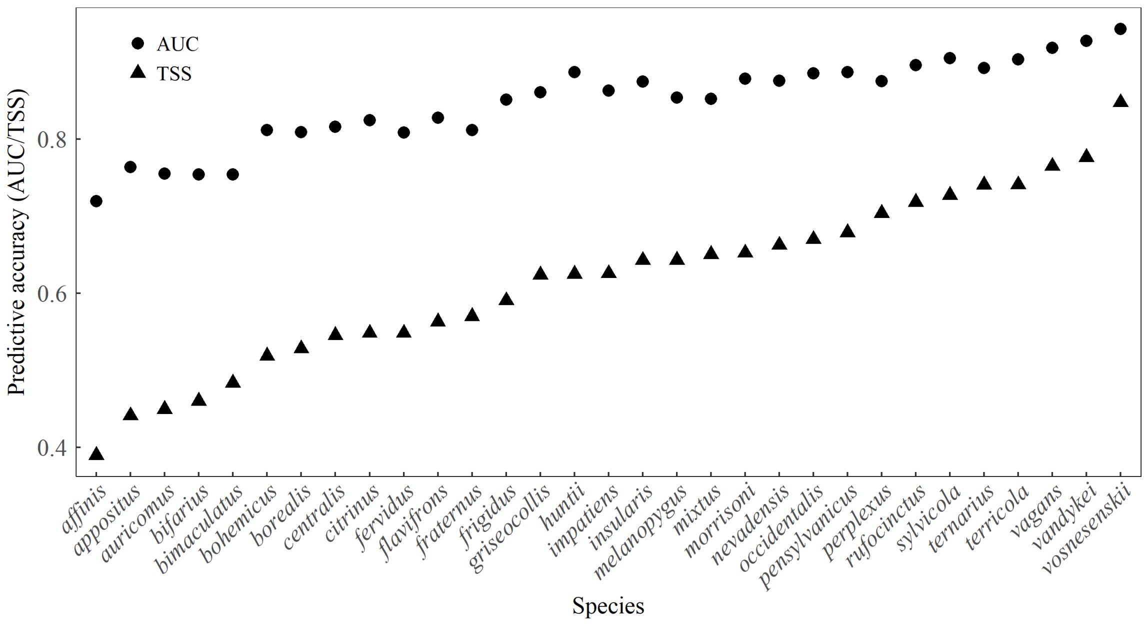

Figure S4: Predictive accuracy of maxent models using the Area Under the Receiver Operator Characteristic Curve (AUC)[3], and the True Skill Statistic (TSS)[4], ordered by TSS. AUC > 0.75 and TSS > 0.4 indicate meaningful models that can be used for analysis.

References

1. Hijmans, R. J., Cameron, S. E., Parra, J. L., Jones, P. G. & Jarvis, A. Very high resolution interpolated climate surfaces for global land areas. *Int. J. Climatol.* **25,** 1965–1978 (2005).

2. IPCC. Climate Change 2014: Synthesis Report. Contribution of Working Groups I, II and III to the Fifth Assessment Report of the Intergovernmental Panel on Climate Change. 151 (2014). doi:10.1017/CBO9781107415324.004

3. Hanley, A. J. & McNeil, J. B. The Meaning and Use of the Area under a Receiver Operating Characteristic (ROC) Curve. *Radiology* **143,** 29–36 (1982).

4. Allouche, O., Tsoar, A. & Kadmon, R. Assessing the accuracy of species distribution models: Prevalence, kappa and the true skill statistic (TSS). *J. Appl. Ecol.* **43,** 1223–1232 (2006).
